# (*E*)-3-(4-Cyclo­hexyl-3-fluoro­benzyl­idene)chroman-4-one

**DOI:** 10.1107/S1600536812024336

**Published:** 2012-05-31

**Authors:** Kaalin Gopaul, Mahidansha M. Shaikh, Neil A. Koorbanally, Deresh Ramjugernath, Bernard Omondi

**Affiliations:** a School of Chemistry and Physics, University of KwaZulu-Natal, Private Bag X54001, Durban 4000, South Africa; b School of Engineering, University of KwaZulu-Natal, Private Bag X54001, Durban 4000, South Africa

## Abstract

The title compound, C_22_H_21_FO_2_, exhibits substitutional disorder of the F atom and a H atom in the asymmetric unit with different occupancies, the refined F:H ratio being 0.80 (2):0.20 (2). The dihedral angle between the fluorinated benzene ring and the benzene ring of the chromanone system is 37.30°. There are two relatively high residual electron-density peaks associated with the disorder.

## Related literature

For the preparation, see: Shaikh *et al.* (2011 ▶). For related structures, see: Gopaul *et al.* (2012 ▶); Marx *et al.* (2008 ▶); Suresh *et al.* (2007 ▶). For the biological activity of this class of compound, see: du Toit *et al.* (2010 ▶). This compound may undergo chemical conversion into the (*E*)- and (*Z*)-isomers, see: Kirkiacharian *et al.* (1984 ▶).
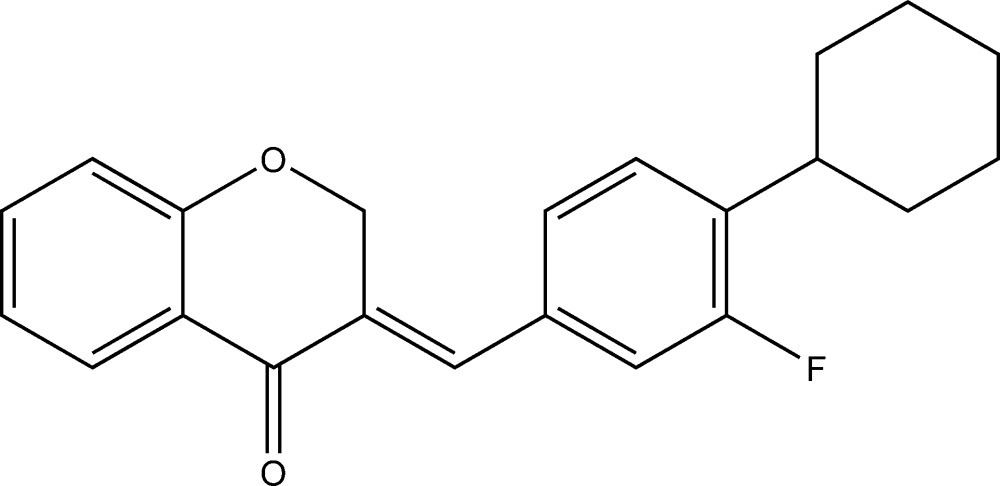


## Experimental

### 

#### Crystal data

C_22_H_21_FO_2_
*M* *_r_* = 336.39
Triclinic, 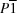

*a* = 6.8351 (1) Å
*b* = 8.1483 (2) Å
*c* = 15.7931 (3) Å
α = 76.661 (1)°
β = 81.769 (1)°
γ = 75.287 (1)°
*V* = 824.45 (3) Å^3^
*Z* = 2
Mo *K*α radiation
μ = 0.09 mm^−1^
*T* = 446 K
0.34 × 0.33 × 0.19 mm

#### Data collection

Bruker SMART APEXII CCD diffractometer
Absorption correction: multi-scan (*SADABS*; Bruker, 2008 ▶) *T*_min_ = 0.969, *T*_max_ = 0.983
18883 measured reflections
4104 independent reflections
3637 reflections with *I* > 2σ(*I*)
*R*_int_ = 0.029

#### Refinement

*R*[*F* ^2^ > 2σ(*F* ^2^)] = 0.058
*wR*(*F* ^2^) = 0.173
*S* = 1.05
4104 reflections
233 parameters
21 restraints
H-atom parameters constrained
Δρ_max_ = 0.96 e Å^−3^
Δρ_min_ = −1.05 e Å^−3^

### 

Data collection: *APEX2* (Bruker, 2008 ▶); cell refinement: *SAINT-Plus* (Bruker, 2008 ▶); data reduction: *SAINT-Plus* and *XPREP* (Bruker, 2008 ▶); program(s) used to solve structure: *SHELXS97* (Sheldrick, 2008 ▶); program(s) used to refine structure: *SHELXL97* (Sheldrick, 2008 ▶); molecular graphics: *ORTEP-3* (Farrugia, 1997 ▶); software used to prepare material for publication: *WinGX* (Farrugia, 1999 ▶).

## Supplementary Material

Crystal structure: contains datablock(s) global, I. DOI: 10.1107/S1600536812024336/fj2556sup1.cif

Structure factors: contains datablock(s) I. DOI: 10.1107/S1600536812024336/fj2556Isup2.hkl

Additional supplementary materials:  crystallographic information; 3D view; checkCIF report

## Figures and Tables

**Fig. 1.**
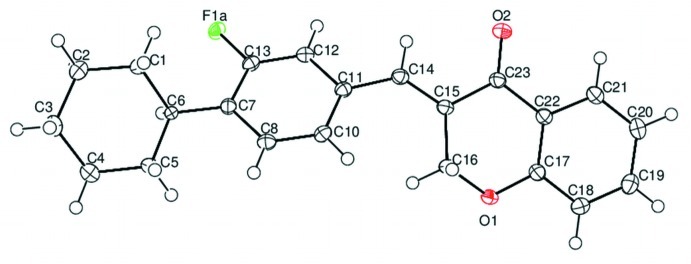
The asymmetric unit of the title compound with displacement ellipsoids drawn at the 50% probability level. The atoms F1a/F1b and H-8/H-13 are disordered and only F1a and H-8 are shown.

## supplementary crystallographic information

### Comment

The title compound, 3-(4-cyclohexyl-3-fluorobenzylidene)chroman-4-one, belongs to the homoisoflavonoid class of compounds, which are *α*,*β* unsaturated carbonyl compounds containing two aromatic rings. They are a group of naturally occurring molecules that are structurally related to isoflavonoids but differ by containing one more carbon atoms (Kirkiacharian *et al.*, 1984). This compound may undergo chemical conversion into the (E)- and (*Z*)-isomers (Kirkiacharian *et al.*, 1984). The 3-benzylidene-4-chromanones have been shown to display a wide range of biological activities (du Toit *et al.*, 2010). The most commonly used procedure for the synthesis of homoisoflavonoids involves the condensation of chroman-4-one with an aromatic aldehyde in the presence of an acidic or basic catalyst (Shaikh *et al.*, 2011). We have recently been involved in the synthesis and characterization of fluorinated homoisoflavonoids in the search for lead pharmaceuticals (Gopaul *et al.*, 2012).

In the molecular structure, the dihedral angle between the fluorinated benzene moiety and the benzene ring of the chromanone moiety is 37.30°. The cyclohexane moiety on the fluorinated benzene ring is attached to the least sterically hindered *para*-position of the phenyl ring and adopts a chair confirmation.

### Experimental

A mixture of chroman-4-one (1.00 g, 6.749 mmol), 3,4-difluorobenzaldehyde (1.15 g, 8.099 mmol) and 10–15 drops of piperidine in cyclohexane was heated at 80°C for 24 hrs. The reaction mixture was monitored for completion by thin layer chromatography. Upon completion, the reaction mixture was cooled, diluted with water and neutralized using 10% HCl. The reaction mixture was extracted with ethyl acetate (3 × 30 ml). The ethyl acetate layers were combined, washed with brine (20 ml), water (2 × 10 ml) and dried over anhydrous magnesium sulfate. The solvent was reduced and the compound purified by column chromatography using silica gel (Merck 9385, 40–63 *µ*m particle size) with a mobile phase of 2% ethyl acetate in hexane to yield the title compound.

^1^H NMR: *δ* (p.p.m.): 1.65 (7*H*, m, H-5''- H-11''), 3.12 (4*H*, m, H-1''- H-4''), 5.36 (2*H*, d, *J*=1.68 Hz, H-2), 7.06–6.92 (5*H*, m, H-2', H-5', H-6', H-6, H-8), 7.46 (1*H*, ddd, *J*=8.48, 6.96, 1.52 Hz, H-7), 7.74 (1*H*, s, H-9), 8.00 (1*H*, dd, *J*=7.84, 1.44 Hz, H-5). ^13^C NMR: δ (p.p.m.): 181.9, 160.9, 154.7 (*J*=246.6 Hz), 142.3 (*J*=8.24 Hz), 136.4 (*J*=1.94 Hz), 135.70, 129.2, 127.9, 127.3 (*J*=2.71 Hz), 122.1, 121.9, 118.7 (*J*=3.94 Hz), 117.83, 117.79 (*J*=22.02 Hz), 67.7, 51.5 (*J*=4.19 Hz), 25.9, 24.2.

### Crystal data

**Table:**

C_22_H_21_FO_2_	*Z* = 2
*M**_r_* = 336.39	*F*(000) = 356
Triclinic, *P*1	*D*_x_ = 1.355 Mg m^−^^3^
Hall symbol: -P 1	Mo *K*α radiation, λ = 0.71073 Å
*a* = 6.8351 (1) Å	Cell parameters from 18935 reflections
*b* = 8.1483 (2) Å	θ = 2.6–28.5°
*c* = 15.7931 (3) Å	µ = 0.09 mm^−^^1^
α = 76.661 (1)°	*T* = 446 K
β = 81.769 (1)°	Block, yellow
γ = 75.287 (1)°	0.34 × 0.33 × 0.19 mm
*V* = 824.45 (3) Å^3^	

### Data collection

**Table:**

Bruker SMART APEXII CCD diffractometer	3637 reflections with *I* > 2σ(*I*)
Graphite monochromator	*R*_int_ = 0.029
φ and ω scans	θ_max_ = 28.5°, θ_min_ = 2.6°
Absorption correction: multi-scan (*SADABS*; Bruker, 2008)	*h* = −9→9
*T*_min_ = 0.969, *T*_max_ = 0.983	*k* = −8→10
18883 measured reflections	*l* = −21→21
4104 independent reflections	

### Refinement

**Table:**

Refinement on *F*^2^	Primary atom site location: structure-invariant direct methods
Least-squares matrix: full	Secondary atom site location: difference Fourier map
*R*[*F*^2^ > 2σ(*F*^2^)] = 0.058	Hydrogen site location: inferred from neighbouring sites
*wR*(*F*^2^) = 0.173	H-atom parameters constrained
*S* = 1.05	*w* = 1/[σ^2^(*F*_o_^2^) + (0.0944*P*)^2^ + 0.7446*P*] where *P* = (*F*_o_^2^ + 2*F*_c_^2^)/3
4104 reflections	(Δ/σ)_max_ < 0.001
233 parameters	Δρ_max_ = 0.96 e Å^−^^3^
21 restraints	Δρ_min_ = −1.05 e Å^−^^3^

### Special details

**Table:**

Experimental. Carbon-bound H-atoms were placed in calculated positions [C—H = 0.97 Å for Methylene H atoms, 0.98 Å for methine and 0.93 Å for aromatic H atoms; *U*_iso_(H) = 1.2*U*_eq_(C)] and were included in the refinement in the riding model approximation. Disorder: Disorder was found for the F– and H-atoms, which is not an uncommon situation. The disorder was modelled for F– and H– atoms (80:30) using PART instructions and the total occupancy at each atom site was kept as 1 during the refinement by means of a SUMFIX constraint. The F-atoms involved in disorder were modelled with anisotropic thermal parameters.
Geometry. All e.s.d.'s (except the e.s.d. in the dihedral angle between two l.s. planes) are estimated using the full covariance matrix. The cell e.s.d.'s are taken into account individually in the estimation of e.s.d.'s in distances, angles and torsion angles; correlations between e.s.d.'s in cell parameters are only used when they are defined by crystal symmetry. An approximate (isotropic) treatment of cell e.s.d.'s is used for estimating e.s.d.'s involving l.s. planes.
Refinement. Refinement of *F*^2^ against ALL reflections. The weighted *R*-factor *wR* and goodness of fit *S* are based on *F*^2^, conventional *R*-factors *R* are based on *F*, with *F* set to zero for negative *F*^2^. The threshold expression of *F*^2^ > σ(*F*^2^) is used only for calculating *R*-factors(gt) *etc*. and is not relevant to the choice of reflections for refinement. *R*-factors based on *F*^2^ are statistically about twice as large as those based on *F*, and *R*- factors based on ALL data will be even larger.

### Fractional atomic coordinates and isotropic or equivalent isotropic displacement parameters (Å^2^)

**Table:**

	*x*	*y*	*z*	*U*_iso_*/*U*_eq_	Occ. (<1)
C1	0.2972 (3)	0.3337 (3)	0.18026 (11)	0.0240 (4)	
H1A	0.4375	0.2691	0.1775	0.029*	
H1B	0.297	0.4558	0.1702	0.029*	
C2	0.1930 (3)	0.3034 (3)	0.10912 (11)	0.0256 (4)	
H2A	0.215	0.1798	0.1126	0.031*	
H2B	0.2531	0.3532	0.0527	0.031*	
C3	−0.0336 (3)	0.3826 (3)	0.11618 (12)	0.0269 (4)	
H3A	−0.0566	0.5079	0.1001	0.032*	
H3B	−0.0978	0.3443	0.0757	0.032*	
C4	−0.1284 (3)	0.3313 (3)	0.20779 (12)	0.0276 (4)	
H4A	−0.129	0.2093	0.2193	0.033*	
H4B	−0.2683	0.3964	0.2122	0.033*	
C5	−0.0171 (2)	0.3636 (3)	0.27620 (11)	0.0252 (4)	
H5A	−0.0337	0.4874	0.2704	0.03*	
H5B	−0.0759	0.3186	0.3338	0.03*	
C6	0.1987 (2)	0.28131 (19)	0.26771 (9)	0.0131 (3)	
H6	0.1978	0.1604	0.2696	0.016*	
C7	0.3101 (2)	0.2672 (2)	0.33810 (10)	0.0158 (3)	
C8	0.2403 (2)	0.3561 (2)	0.40676 (10)	0.0181 (3)	
H8	0.1123	0.4306	0.4063	0.022*	0.803 (4)
F1B	0.0633 (2)	0.4701 (2)	0.41034 (10)	0.0309 (17)	0.197 (4)
C10	0.3560 (2)	0.3365 (2)	0.47542 (10)	0.0178 (3)	
H10	0.3038	0.3982	0.5195	0.021*	
C11	0.5499 (2)	0.22563 (19)	0.47953 (10)	0.0160 (3)	
C12	0.6215 (2)	0.13448 (19)	0.41124 (10)	0.0164 (3)	
H12	0.7496	0.0599	0.4114	0.02*	
C13	0.5036 (2)	0.15504 (19)	0.34448 (10)	0.0161 (3)	
H13	0.5545	0.0913	0.3011	0.019*	0.197 (4)
F1A	0.57542 (18)	0.05863 (15)	0.28206 (8)	0.0190 (4)	0.803 (4)
C14	0.6861 (2)	0.1993 (2)	0.54697 (10)	0.0170 (3)	
H14	0.8215	0.1522	0.5311	0.02*	
C15	0.6495 (2)	0.23152 (19)	0.62847 (10)	0.0159 (3)	
C16	0.4451 (2)	0.3066 (2)	0.67092 (10)	0.0183 (3)	
H16A	0.4237	0.4318	0.6582	0.022*	
H16B	0.3413	0.2779	0.6448	0.022*	
C17	0.5798 (2)	0.2205 (2)	0.81068 (10)	0.0180 (3)	
C18	0.5371 (3)	0.2086 (2)	0.90100 (11)	0.0231 (3)	
H18	0.404	0.2192	0.9261	0.028*	
C19	0.6940 (3)	0.1809 (2)	0.95265 (11)	0.0239 (4)	
H19	0.6657	0.1728	1.0127	0.029*	
C20	0.8948 (3)	0.1650 (2)	0.91576 (11)	0.0223 (3)	
H20	0.9991	0.1495	0.9508	0.027*	
C21	0.9374 (2)	0.1725 (2)	0.82692 (11)	0.0196 (3)	
H21	1.0712	0.159	0.8025	0.023*	
C22	0.7803 (2)	0.20013 (19)	0.77313 (10)	0.0165 (3)	
C23	0.8274 (2)	0.1979 (2)	0.67917 (10)	0.0180 (3)	
O1	0.41878 (17)	0.24763 (18)	0.76439 (8)	0.0230 (3)	
O2	1.00278 (19)	0.1688 (2)	0.64622 (9)	0.0303 (3)	

### Atomic displacement parameters (Å^2^)

**Table:**

	*U*^11^	*U*^22^	*U*^33^	*U*^12^	*U*^13^	*U*^23^
C1	0.0204 (8)	0.0353 (9)	0.0172 (7)	−0.0094 (7)	0.0026 (6)	−0.0064 (6)
C2	0.0245 (8)	0.0333 (9)	0.0185 (8)	−0.0042 (7)	−0.0007 (6)	−0.0081 (7)
C3	0.0239 (8)	0.0373 (10)	0.0222 (8)	−0.0083 (7)	−0.0058 (6)	−0.0078 (7)
C4	0.0189 (8)	0.0393 (10)	0.0255 (9)	−0.0077 (7)	−0.0028 (6)	−0.0070 (7)
C5	0.0147 (7)	0.0397 (10)	0.0221 (8)	−0.0066 (6)	0.0001 (6)	−0.0085 (7)
C6	0.0111 (6)	0.0177 (7)	0.0106 (6)	−0.0035 (5)	−0.0004 (5)	−0.0031 (5)
C7	0.0165 (7)	0.0173 (7)	0.0144 (6)	−0.0067 (5)	−0.0008 (5)	−0.0020 (5)
C8	0.0161 (7)	0.0170 (7)	0.0197 (7)	−0.0019 (5)	0.0003 (5)	−0.0042 (6)
F1B	0.023 (3)	0.034 (3)	0.032 (3)	0.009 (2)	−0.007 (2)	−0.014 (2)
C10	0.0189 (7)	0.0172 (7)	0.0166 (7)	−0.0026 (6)	0.0006 (5)	−0.0053 (5)
C11	0.0168 (7)	0.0154 (7)	0.0160 (7)	−0.0054 (5)	0.0006 (5)	−0.0029 (5)
C12	0.0154 (7)	0.0151 (7)	0.0181 (7)	−0.0034 (5)	0.0007 (5)	−0.0034 (5)
C13	0.0188 (7)	0.0143 (7)	0.0158 (7)	−0.0049 (5)	0.0017 (5)	−0.0047 (5)
F1A	0.0210 (6)	0.0186 (6)	0.0182 (6)	−0.0015 (4)	−0.0006 (4)	−0.0093 (4)
C14	0.0151 (7)	0.0161 (7)	0.0194 (7)	−0.0024 (5)	−0.0004 (5)	−0.0051 (5)
C15	0.0133 (6)	0.0159 (7)	0.0184 (7)	−0.0031 (5)	−0.0001 (5)	−0.0045 (5)
C16	0.0137 (7)	0.0242 (8)	0.0158 (7)	−0.0025 (6)	0.0006 (5)	−0.0047 (6)
C17	0.0168 (7)	0.0190 (7)	0.0189 (7)	−0.0044 (5)	−0.0020 (6)	−0.0046 (6)
C18	0.0197 (7)	0.0292 (9)	0.0197 (8)	−0.0053 (6)	0.0010 (6)	−0.0057 (6)
C19	0.0283 (9)	0.0262 (8)	0.0180 (7)	−0.0070 (7)	−0.0029 (6)	−0.0046 (6)
C20	0.0240 (8)	0.0218 (8)	0.0232 (8)	−0.0064 (6)	−0.0082 (6)	−0.0037 (6)
C21	0.0172 (7)	0.0192 (7)	0.0234 (8)	−0.0046 (6)	−0.0035 (6)	−0.0050 (6)
C22	0.0155 (7)	0.0153 (7)	0.0190 (7)	−0.0029 (5)	−0.0020 (5)	−0.0043 (5)
C23	0.0142 (7)	0.0199 (7)	0.0205 (7)	−0.0025 (5)	−0.0007 (5)	−0.0076 (6)
O1	0.0133 (5)	0.0381 (7)	0.0171 (6)	−0.0064 (5)	0.0002 (4)	−0.0048 (5)
O2	0.0140 (6)	0.0507 (9)	0.0273 (7)	−0.0028 (5)	0.0010 (5)	−0.0172 (6)

### Geometric parameters (Å, º)

**Table:**

C1—C6	1.467 (2)	C11—C14	1.457 (2)
C1—C2	1.509 (2)	C12—C13	1.372 (2)
C1—H1A	0.97	C12—H12	0.93
C1—H1B	0.97	C13—F1A	1.3611 (18)
C2—C3	1.518 (2)	C13—H13	0.93
C2—H2A	0.97	C14—C15	1.349 (2)
C2—H2B	0.97	C14—H14	0.93
C3—C4	1.509 (3)	C15—C23	1.484 (2)
C3—H3A	0.97	C15—C16	1.504 (2)
C3—H3B	0.97	C16—O1	1.4433 (19)
C4—C5	1.511 (2)	C16—H16A	0.97
C4—H4A	0.97	C16—H16B	0.97
C4—H4B	0.97	C17—O1	1.3517 (19)
C5—C6	1.458 (2)	C17—C22	1.398 (2)
C5—H5A	0.97	C17—C18	1.400 (2)
C5—H5B	0.97	C18—C19	1.382 (2)
C6—C7	1.403 (2)	C18—H18	0.93
C6—H6	0.98	C19—C20	1.399 (3)
C7—C8	1.402 (2)	C19—H19	0.93
C7—C13	1.406 (2)	C20—C21	1.382 (2)
C8—F1B	1.3282	C20—H20	0.93
C8—C10	1.389 (2)	C21—C22	1.405 (2)
C8—H8	0.93	C21—H21	0.93
C10—C11	1.402 (2)	C22—C23	1.476 (2)
C10—H10	0.93	C23—O2	1.224 (2)
C11—C12	1.410 (2)		
			
C6—C1—C2	112.53 (14)	C11—C10—H10	119.4
C6—C1—H1A	109.1	C10—C11—C12	117.11 (14)
C2—C1—H1A	109.1	C10—C11—C14	125.82 (14)
C6—C1—H1B	109.1	C12—C11—C14	117.04 (14)
C2—C1—H1B	109.1	C13—C12—C11	120.46 (14)
H1A—C1—H1B	107.8	C13—C12—H12	119.8
C1—C2—C3	112.13 (14)	C11—C12—H12	119.8
C1—C2—H2A	109.2	F1A—C13—C12	118.33 (14)
C3—C2—H2A	109.2	F1A—C13—C7	118.06 (14)
C1—C2—H2B	109.2	C12—C13—C7	123.59 (14)
C3—C2—H2B	109.2	C12—C13—H13	118.2
H2A—C2—H2B	107.9	C7—C13—H13	118.2
C4—C3—C2	111.15 (15)	C15—C14—C11	131.10 (14)
C4—C3—H3A	109.4	C15—C14—H14	114.5
C2—C3—H3A	109.4	C11—C14—H14	114.5
C4—C3—H3B	109.4	C14—C15—C23	117.23 (14)
C2—C3—H3B	109.4	C14—C15—C16	125.62 (14)
H3A—C3—H3B	108	C23—C15—C16	117.08 (13)
C3—C4—C5	112.63 (15)	O1—C16—C15	114.82 (13)
C3—C4—H4A	109.1	O1—C16—H16A	108.6
C5—C4—H4A	109.1	C15—C16—H16A	108.6
C3—C4—H4B	109.1	O1—C16—H16B	108.6
C5—C4—H4B	109.1	C15—C16—H16B	108.6
H4A—C4—H4B	107.8	H16A—C16—H16B	107.5
C6—C5—C4	111.53 (15)	O1—C17—C22	123.41 (14)
C6—C5—H5A	109.3	O1—C17—C18	116.25 (14)
C4—C5—H5A	109.3	C22—C17—C18	120.32 (15)
C6—C5—H5B	109.3	C19—C18—C17	119.57 (15)
C4—C5—H5B	109.3	C19—C18—H18	120.2
H5A—C5—H5B	108	C17—C18—H18	120.2
C7—C6—C5	117.07 (13)	C18—C19—C20	120.78 (16)
C7—C6—C1	116.12 (13)	C18—C19—H19	119.6
C5—C6—C1	112.93 (13)	C20—C19—H19	119.6
C7—C6—H6	102.6	C21—C20—C19	119.60 (15)
C5—C6—H6	102.6	C21—C20—H20	120.2
C1—C6—H6	102.6	C19—C20—H20	120.2
C8—C7—C6	124.42 (14)	C20—C21—C22	120.61 (15)
C8—C7—C13	115.31 (14)	C20—C21—H21	119.7
C6—C7—C13	120.25 (14)	C22—C21—H21	119.7
F1B—C8—C10	114.28 (9)	C17—C22—C21	119.08 (14)
F1B—C8—C7	123.45 (9)	C17—C22—C23	120.42 (14)
C10—C8—C7	122.22 (14)	C21—C22—C23	120.41 (14)
C10—C8—H8	118.9	O2—C23—C22	121.42 (15)
C7—C8—H8	118.9	O2—C23—C15	122.84 (15)
C8—C10—C11	121.29 (14)	C22—C23—C15	115.73 (13)
C8—C10—H10	119.4	C17—O1—C16	118.16 (12)
			
C6—C1—C2—C3	51.8 (2)	C10—C11—C14—C15	19.5 (3)
C1—C2—C3—C4	−49.7 (2)	C12—C11—C14—C15	−162.10 (17)
C2—C3—C4—C5	50.9 (2)	C11—C14—C15—C23	−176.66 (15)
C3—C4—C5—C6	−53.7 (2)	C11—C14—C15—C16	0.3 (3)
C4—C5—C6—C7	−165.85 (15)	C14—C15—C16—O1	148.36 (16)
C4—C5—C6—C1	55.3 (2)	C23—C15—C16—O1	−34.7 (2)
C2—C1—C6—C7	165.83 (15)	O1—C17—C18—C19	179.99 (15)
C2—C1—C6—C5	−54.9 (2)	C22—C17—C18—C19	1.7 (3)
C5—C6—C7—C8	−13.6 (2)	C17—C18—C19—C20	0.1 (3)
C1—C6—C7—C8	123.94 (17)	C18—C19—C20—C21	−1.7 (3)
C5—C6—C7—C13	164.76 (15)	C19—C20—C21—C22	1.6 (2)
C1—C6—C7—C13	−57.7 (2)	O1—C17—C22—C21	−179.98 (14)
C6—C7—C8—F1B	−3.22 (19)	C18—C17—C22—C21	−1.8 (2)
C13—C7—C8—F1B	178.34 (9)	O1—C17—C22—C23	−3.4 (2)
C6—C7—C8—C10	179.45 (15)	C18—C17—C22—C23	174.79 (15)
C13—C7—C8—C10	1.0 (2)	C20—C21—C22—C17	0.2 (2)
F1B—C8—C10—C11	−177.69 (11)	C20—C21—C22—C23	−176.43 (15)
C7—C8—C10—C11	−0.1 (2)	C17—C22—C23—O2	−175.13 (16)
C8—C10—C11—C12	−0.3 (2)	C21—C22—C23—O2	1.4 (2)
C8—C10—C11—C14	178.12 (14)	C17—C22—C23—C15	4.4 (2)
C10—C11—C12—C13	−0.2 (2)	C21—C22—C23—C15	−179.02 (14)
C14—C11—C12—C13	−178.77 (14)	C14—C15—C23—O2	11.4 (2)
C11—C12—C13—F1A	−177.15 (13)	C16—C15—C23—O2	−165.74 (16)
C11—C12—C13—C7	1.2 (2)	C14—C15—C23—C22	−168.11 (14)
C8—C7—C13—F1A	176.79 (13)	C16—C15—C23—C22	14.7 (2)
C6—C7—C13—F1A	−1.7 (2)	C22—C17—O1—C16	−18.0 (2)
C8—C7—C13—C12	−1.6 (2)	C18—C17—O1—C16	163.70 (15)
C6—C7—C13—C12	179.93 (14)	C15—C16—O1—C17	36.5 (2)
